# Pre-Implantation Effects of Progesterone Administration on Ovarian Angiogenesis after Ovarian Stimulation: A Histological, Hormonal, and Molecular Analysis

**DOI:** 10.5935/1518-0557.20190076

**Published:** 2020

**Authors:** Leila Narimani, Nasim Beigi Boroujeni, Mohammadreza Gholami, Khatereh Anbari, Seyyed Ezatollah Rafiee Alavi, Seyyed Amir Yasin Ahmadi, Mandana Beigi Boroujeni

**Affiliations:** 1 Department of Anatomical Sciences, Lorestan University of Medical Sciences, Khorramabad, Iran; 2 Razi Herbal Medicines Research Center, Lorestan University of Medical Sciences, Khorramabad, Iran; 3 Department of Anatomical Sciences, Kermanshah University of Medical Sciences, Kermanshah, Iran; 4 Social Determinants of Health Research Center, Lorestan University of Medical Sciences, Khorramabad, Iran; 5 Department of Pathology, Lorestan University of Medical Sciences, Khoramabad, Iran; 6 Pediatric Growth and Development Research Center, Institute of Endocrinology and Metabolism, Iran University of Medical Sciences, Tehran, Iran

**Keywords:** progesterone, ovary, angiogenesis, pre-implantation, ovarian stimulation, corpus luteum

## Abstract

**Objective:**

Progesterone (P4) is known to directly affect ovarian tissue angiogenesis. The present study was designed to show how P4 affects ovarian angiogenesis in hormonal, histological, and molecular levels.

**Methods:**

Fifteen adult female NMRI mice were divided into three groups: Control Group; Case Group I (ovarian stimulation alone); and Case Group II (ovarian stimulation followed by P4 administration). Blood and ovarian tissue samples were assessed for hormonal, histological, and molecular alterations. Gene expression for ovarian vascular endothelium growth factor (VEGF) and hypoxia-inducible factor-1 alpha (HIF-1α) was analyzed using real-time PCR.

**Results:**

Ovarian hormone levels were increased in the case groups compared with the control group (*p*<0.05). Quantitative corpus luteum parameters were increased in the case groups compared with the control group (*p*<0.05). Quantitative ovarian vascular parameters were significantly different in the case groups compared with the control group. Gene expression analyses shows that the mice in Case Group I had higher levels of ovarian VEGF expression than the mice in the control group (*p*<0.05). No significant difference in gene expression was observed for HIF-1ɑ.

**Conclusion:**

Treatment with P4 after ovarian stimulation enhanced ovarian angiogenesis by increasing hormone levels and causing significant structural changes.

## INTRODUCTION

 In recent decades, assisted reproduction technology (ART) has led to increased pregnancy rates. Regulated ovarian stimulation causes the development of multiple follicles and angiogenesis activation (Artini *et al*., 1998; Gad *et al*., 2011; Sibug *et al*., 2002). Ovarian hormones have been used to induce angiogenesis *in vitro* and *in vivo* (Wen *et al*., 2009). Research found that estrogen increases vascular permeability and inhibits angiogenesis, while progesterone stimulates angiogenesis and produces lesser effects on vascular permeability. Little is known about the mechanisms by which ovarian steroids affect angiogenesis (Daikoku *et al*., 2003).

Progesterone (P4) is a steroid hormone with a key role in ovulation. P4 regulates granulosa and luteal cell function via numerous intraovarian actions. Many receptors and signal transduction pathways have been associated with P4 mechanisms, but they are not completely known. These mechanisms ultimately result in the inhibition of mitosis and apoptosis in granulosa and luteal cells (Peluso, 2006).

Despite growing research in the area, the pre-implantation effects of P4 on ovarian angiogenesis after stimulation are still unclear. This study was designed to show how P4 administration after ovarian stimulation affects ovarian angiogenesis in hormonal, histological, and molecular levels.

## MATERIAL AND METHODS

### Animals

A total of 15 adult female NMRI mice (6-10 weeks of age) were used in this study. The mice were divided into three groups of five subjects each, as follows: Control Group; Case Group I (ovarian stimulation alone); and Case Group II (ovarian stimulation followed by P4 administration). Ethical principles regarding the use of animals in research were observed. The mice in case groups underwent ovulation induction. Pseudopregnancy was induced in all three groups. Ovulation induction was performed with an intraperitoneal injection of human menopausal gonadotropin (HMG) (10 IU) followed by an injection of human chorionic gonadotropin (HCG) (10 IU) 48 hours later. P4 (1mg/mouse) was injected daily in the subjects in case group II after ovulation induction and after pseudopregnancy induction. The hearts of the mice were excised before implantation time to assess the cardiac effects derived from hormonal changes. Ovarian tissue was collected to assess histological and molecular changes.

### Tissue preparation

After tissue preparation, the sections stained with Hematoxylin and Eosin according to standard histology procedures were analyzed on software package Motic Images Plus 2.0 for quantitative parameters including corpus luteum number, corpus luteum area, corpus luteum vascular area, and ovarian vessel area on a light microscope. A probe was used on tissue sections to calculate vascular density. Eight to 12 sections were selected from each specimen and five random fields of view at 400x magnification were assessed for each section.

### Hormonal assessment

Cardiac blood samples were taken from the studied animals and tested with ELISA kits to measure estrogen and progesterone levels (Estradiol Elisa. Cat. No: DCM003-11, Dia Metra Italy) (Progesterone Elisa. Cat. No: DCM006-9, Dia Metra Italy).

### RNA extraction and cDNA synthesis

RNA extraction was carried out in accordance with kit manufacturer instructions (Yekta Tajhiz Azma, Cat. No: YT9065). RNA purity (1.8-2) and RNA concentration (180-380ng/µl) were assessed by NanoDrop Spectrophotometer at a wavelength of 260nm. cDNA synthesis was assessed in accordance with kit manufacturer instructions (Sina Clone, Cat. No: RT5201). cDNA purity (1.7) and cDNA concentration (400-500ng/µl) were analyzed with ratio of absorbance at 260nm and 280nm.

### Real-Time PCR

Vascular endothelial growth factor (VEGF) and hypoxia-inducible factor-1 alpha (HIF-1α) gene expression levels were compared with the level of ß-actin expression. Forty cycles were carried out each for 15s at 95ºC, 60s at 60ºC. Control cycles contained the PCR mix without cDNA. Table 1 shows the mouse primer sequences used in real-time PCR.

**Table 1:** Mouse primer sequences used in real-time PCR.

Gene	Primer	Length	Annealing Temperature	Sequence 5′→ 3′	Gene bank code
VEGF	Forward	22	64	GGAGACTCTTCGAGGAGCACTT	NM_001110268.1
VEGF	Reverse	25	62.5	GGCGATTTAGCAGCAGATATAAGAA	NM_001110268.1
HIF-1α	Forward	20	58.4	ACCCATTCCTCATCCGTCAA	NM_017314961.1
HIF-1α	Reverse	20	58.4	ATTGAGCGGCCCAAAAGTTC	NM_017314961.1
β-Actin	Forward	16	58.4	CCGCCACCAGTTCGCC	NM_00739305
β-Actin	Reverse	21	63.3	CTCGTCACCCACATAGGAGTC	NM_00739305

### Statistical analysis

Data analysis was performed on software package SPSS Statistics 19 (IBM, USA). One-way ANOVA and Tukey post-hoc test - or its nonparametric equivalent (Kruskal-Wallis test) - were used. Differences with a *p*≤0.05 were deemed statistically significant. Data from real-time PCR were analyzed on bioinformatics software REST.

## RESULTS

### Histology findings

**Figure 1:**
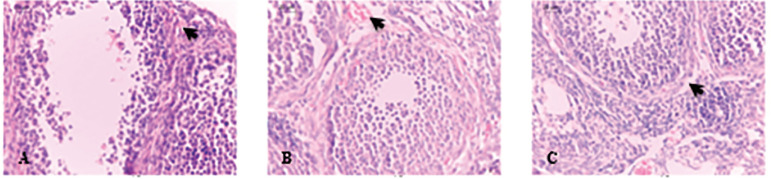
The comparison of quantitative ovarian parameters (Ovarian vascular density and ovarian vessel area) between the control group and case groups (light microscopy, 400× magnification) found a significant difference at pre-implantation time. The arrow shows the ovarian vessels. Control group [A], Ovarian stimulation group [B], and P4-treated group [C].

**Figure 2:**
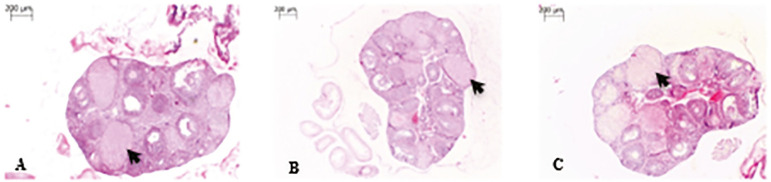
The comparison of quantitative corpus luteum parameters (corpus luteum number, corpus luteum area, and corpus luteum vascular area) between control and case groups (light microscopy, 40× magnification) found a significant difference between groups at pre-implantation time. The arrow shows sites of corpus luteum apoptosis. Control group [A], Ovarian stimulation group [B] and P4-treated group [C].

Our study found significant differences when quantitative ovarian vascular parameters (ovarian vascular density and ovarian vessel area) (Figure 1) and quantitative corpus luteum parameters (corpus luteum number, corpus luteum area and corpus luteum vessel area) (Figure 2) of case and control groups were compared (*p*≤0.05) (Table 2).

**Table 2:** Ovarian structural parameters. Mean ± SD in different groups (µm^2^)

	Control Group	Ovarian Stimulation Group	P4 Treated Group after Ovarian Stimulation
Number of Corpora Lutea	5.1±0.42	10.52±2.74^a^	8.97±0.8^a^
Area of Corpora Lutea	31634.1±7526.1	96359.2±36043.2^a^	92092.9±22186.1^a^
Vascular Area of Corpora Lutea	195.01±7.85	221.04±6.77^a^	219.51±9.15^a^
Vascular Area of Ovaries	198.57±3.81	225.62±3.62^a^	230.49±2.25^a^
Density of Ovarian Vessels	0.034±0.004	0.040±0.001^a^	0.044±0.002^a^

a Significant difference with the control group (*p*≤0.05).

### Hormonal findings

Hormonal assessment indicated that case and control groups had significantly different 17-ß estradiol and P4 levels (*p*≤0.05) (Table 3).

**Table 3:** Mean levels of progesterone (ng/ml) and estrogen (pg/ml) in different groups.

	Control Group	Ovarian Stimulation Group	P4 Treated Group after Ovarian Stimulation
Mean Rank of Progesterone	3	8^a^	13^a^^b^
Estradiol	35.4± 4.97	54.0±11.18^a^	60.6±11.61^a^

a Significant difference with the control group (*p*≤0.05).

b Significant difference with the Case Group І (ovarian stimulation group) (*p*≤0.05).

### Gene expression analysis findings

Case Group I and Control Group VEGF gene expression levels were significantly different (*p*≤0.05). Multifold increases in gene expression levels were seen when Case Group II, Case Group I, and Control Group test results were compared. No significant difference was seen in HIF-1ɑ gene expression levels between the groups, although multifold increases were observed between Case Groups I and II and the Control Group and when Case Groups II and I were compared (Table 4).

**Table 4:** Gene expression analysis with REST 2009 software in different group

Group	Gene	Reaction Efficiency	Expression	Std. Error	95% C.I.	P(H1)	Result
Group I with control	HIF-1a	0.8267	2.189	0.515 - 14.246	0.168 - 18.962	0.266	NS
	VEGF	0.8233	4.161	1.255-15.206	0.738-29.553	0.016	UP*
Group II with control	HIF-1a	0.84	1.314	0.179- 8.920	0.028- 68.576	0.788	NS
	VEGF	0.8108	2.551	0.485-12.369	0.153- 47.442	0.238	NS
Group II with I	HIF-1a	0.7983	0.608	0.054- 4.298	0.019 - 19.654	0.589	NS
	VEGF	0.8358	0.610	0.138- 2.186	0.090- 3.033	0.397	NS

* A significant difference with the control group. (*p*≤0.05). UP: up regulation, NS: non-significant

## DISCUSSION

Ovarian vascular density and ovarian vessel area were significantly different when case and control groups were compared. Although the two parameters were increased in Case Group II *vs*. Case Group I, the difference was not statistically significant. Ovarian tissue and other highly vascularized portions of the female reproductive organs rely on angiogenesis and vascular growth for their development and function (Reynolds *et al*., 2002). Angiogenesis rarely occurs in adulthood, with the exception of the female reproductive organs (Reynolds *et al*., 2002; Wong *et al*., 1997). Ovarian angiogenesis leads to the presentation of gonadotropins, growth factors, oxygen, steroidal precursors, and other elements needed in the development of the follicles and the corpus luteum (Abramovich *et al*., 2006). This study found that increases in estrogen levels caused increases in the rate of angiogenesis, and that elevations in estrogen levels coincided with increases in vascular density and consequent increases in vascular area. Although there was no significant difference in the estrogen levels seen in Case Group II *vs.* Case Group I, the higher levels of estrogen seen in Case Group II were accompanied by increases in vascular area and density. This finding elicits the effect estrogen has on the enhancement of angiogenesis. It seems that the hormone fluctuations induced by ovarian stimulation might result in conditions that favor increased angiogenesis. Estrogen has a direct effect on endothelial cell angiogenesis. By their turn, the capillary plexus that develops during the estrous cycle and angiogenic activity rely on estradiol levels. The precise hormonal mechanisms connected to the formation of new blood vessels and the growth of blood vessels in physiological and pathological conditions have to be further elucidated (Losordo & Isner, 2001). S*alvia officinalis* has been described as a phytoestrogen containing estrogenic components and isoflavonoids (Vitale *et al*., 2013). A recent study indicated that the administration of *Salvia officinalis* extract led to increased ovarian vessel area and vascular density before implantation (Boroujeni & Gholami, 2017). Administration of *Salvia officinalis* extract has been linked to increases in uterine vascular area (Boroujeni *et al*., 2016a).

Our findings showed that P4 treatment after ovarian stimulation might enhance angiogenic parameters including vascular density and vascular area, which indicate a role for P4 in increasing the rate of angiogenesis. Our results were consistent with the results published in a previous study (Boroujeni *et al*., 2016b). However, no significant difference was found for Case Group II. It appears that increases in P4 levels beyond a certain point did not further increase angiogenesis in mice. P4 released from ovarian tissue is known to have proangiogenic effects (Kayisli *et al*., 2004). P4 also causes increases in the uterine vascular density of mice without ovarian tissue, while it has lesser effects on vascular permeability (Ma *et al*., 2001). The administration of P4 has been linked to increases in the rate of angiogenesis (Boroujeni *et al*., 2016b). Our findings demonstrated that P4 administration after ovarian stimulation increased the rate of angiogenesis.

Humans, primates, and rodents treated with human chorionic gonadotropin (hCG) had enhanced ovarian tissue angiogenesis. hCG treatment is known to cause increased endothelial cell migration and vascular bud formation along with enhanced synthesis of proteins such as secretogranin II / (SCG2) and secretoneurin (SN) in the granulosa cells before ovulation (Hannon *et al*., 2018). It appears that hCG treatment increased the rate of angiogenesis in the groups offered ovarian stimulation in our study by increasing local vascular density and vascular area.

Antiangiogenic effects from P4 administration may be caused by excessive increases in P4 levels. The effects of P4 and ovarian stimulation drugs on the rate of angiogenesis and on the epithelium of endometrial glands indicated that gonadotropins stimulate endothelial cell proliferation and capillary density growth. P4 administration after ovarian stimulation with gonadotropins had an inhibitory effect on endothelial cell proliferation (Rashidi *et al*., 2017). Some authors believe that uterine coiled arteriolar system growth is affected by P4 in the secretory phase (Greb *et al*., 1997; Shifren *et al*., 1996). Ongoing P4 administration has also been linked to decreased endometrial micro vascular density (Mönckedieck *et al*., 2009). Estrogen has a role in increasing permeability and angiogenic inhibition, while P4 causes angiogenesis and does not increase vascular permeability as effectively. Nevertheless, the mechanisms by which estradiol regulates angiogenesis are largely unknown (Daikoku *et al*., 2003).

The case and control groups included in our study has significant differences in corpus luteum parameters, including corpus luteum number, corpus luteum area, and corpus luteum vascular area. Case groups I and II were not statistically different in terms of these parameters despite their multifold increases. The results for corpus luteum number and corpus luteum area were in accordance with hormonal findings. Ovulation induction led to increased ovarian hormone levels, the maturation of multiple follicles, and higher corpus luteum numbers when case and control groups were compared. P4 treatment after ovarian stimulation did not produce further increases in corpus luteum number or corpus luteum area in Case Group IІ. Concerning the hypothalamic-pituitary-gonadal axis, excess hormone produces negative feedback effects (Hall, 2015). Increased P4 levels did not yield significant negative feedback effects, but mice treated with P4 had fewer corpora lutea and smaller corpus luteum area. hCG induced pregnancy, increased P4 secretion, and led to excess luteal weight (Davis *et al*., 2003). Therapy with hCG and LH promotes the conversion of small corpora lutea luteum into large corpora lutea (Farin *et al*., 1988). Increased corpus luteum area leads to increases in the number of P4 receptors via intensification of intracellular signaling and on account of enhanced angiogenesis in the corpus luteum in relation to the group treated with P4.

P4 and estrogen receptors in the ovarian tissue of women are concentrated in the theca interna cells, granulosa cells, corpus luteum, superficial epithelium of the ovary, luteal cells, and stroma (Juengel *et al*., 2006; Starczewski *et al*., 2008; Vermeirsch *et al*., 2001). Variable expression of membrane progestin receptors in the corpus luteum during the estrous cycle and first trimester of pregnancy has been reported in the literature. Therefore, P4 may also contribute to corpus luteum function via membrane receptors (Kowalik *et al*., 2018).

The level of angiogenesis in the corpus luteum was higher in Case Group I than in Case Group II mice. In ovarian tissue, angiogenesis was more pronounced in the mice in Case Group II. One of the most important reasons for the differences in the level of angiogenesis level seen in the corpus luteum compared with ovarian tissue between Case Group І and II is the number and scattered distribution of P4 receptors in ovarian tissue. Progesterone and its negative feedback effects may produce these changes in various areas of the ovarian tissue. Considering the results seen in our study, it appears that P4 induced the maturation of the corpus luteum. Consequently, the number of cells and vascularization of corpora lutea decreases in relation to earlier stages of development. As for the existence of more ovarian vessels in Case Group II than in Case Group І, more angiogenesis was observed in the stromal zones and other ovarian sites than in corpora lutea, which led to these differences.

The results of our study on VEGF gene expression indicated significant differences between Case Group I and the Control Group. The results of an in vitro study reported that VEGF mRNA and VEGF proteins were expressed in the granulosa cells of monkey follicles, leading to increased endothelial cell migration and endothelial cell bud formation (Kim *et al*., 2017). Ovarian stimulation associated with P4 apparently led to increased VEGF gene expression and angiogenesis. Several studies found that theca and granulosa cells of the ovarian follicles expressed VEGF mRNA at pre-ovulation time. VEGF mRNA expression is also increased in the granulosa cells of the ovarian tissue of infertile patient treated with ovulation induction methods (Reynolds *et al*., 2002; Wang *et al*., 2002). Histology analysis indicated the existence of a greater number of corpora lutea in the case groups, which caused increased VEGF gene expression. VEGF has significant roles in ovulation and egg development, selection, and maturity. It also plays an important role on the maintenance of corpus luteum function. The rate of VEGF gene expression is dependent on gonadotropin hormones, which affect the selection of dominant ovarian follicles. VEGF gene expression increases in a pre-ovulatory follicle. Angiogenesis starts in the developing ovarian follicles before the differentiation of theca cells, and is a key element in folliculogenesis and ovulation (Dong & Cheng, 2003). VEGF gene expression was increased in Case Group II compared with the Control Group, but lower in Case Group II than in Case Group I. Histology analysis found that corpus luteum number and corpus luteum area were decreased in Case Group II compared with Case Group I, possibly due to lower levels of VEGF gene expression in Case Group II. Lower levels of angiogenic factors were therefore present, since they are produced in corpora lutea. Furthermore, VEGF gene expression may have reached the VEGF protein expression level at pre-implantation time and a decline in gene expression may have occurred for this reason. Excess P4 leads to hormonal balance disturbances and resulted in decreased VEGF gene expression in Case Group II compared with Case Group І. The differences in gene expression and level of angiogenesis may have been caused by various factors present in the case groups, including hormone levels, number of receptors in relation to corpus luteum number and area, to name a few.

Angiogenic factor VEGF-A is produced in the corpora lutea and ovarian follicles. VEGF-A gene expression is dependent on follicle size. In addition, ovarian VEGF gene expression is increased in the granulosa and theca cells (Abramovich *et al*., 2006). VEGF is also a vital mediator for endothelial cell proliferation, luteal vascularization, and P4 production in the corpora lutea of rodents (Galvão *et al*., 2013). It also plays an important role on the vascular growth at primary stage (Matsumoto *et al*., 2002). VEGF gene expression and its receptors in luteal and endothelial cells act as an autocrine factor to regulate angiogenesis and corpus luteum vascular permeability. VEGF participates in the maintenance of corpus luteum function by increasing capillary permeability and aiding in the transport of cholesterol into the corpus luteum to produce P4 (Dong & Cheng, 2003). Our data indicated an angiogenic role of ovarian stimulation over P4 levels in VEGF gene expression, although many other factors also affect angiogenesis. VEGF gene expression is controlled with gonadotropins in pre-ovulatory follicles, but the role of gonadotropins is limited in mature follicles. A recent study found that other paracrine factors regulate VEGF gene expression in developing follicles (Taylor *et al*., 2004). The results of the present study found that HIF-1ɑ gene expression was increased in case groups compared with the control group and greater - albeit not significantly - in Case Group I than in Case Group II. Oxygen concentration is reduced in the follicular fluid during oocyte maturation (Nishimura & Okuda, 2015). Therefore, released follicles are affected by hypoxia after ovarian stimulation due to bleeding and immature vascularization (Fadhillah *et al*., 2017). It should be noted that oxygen concentration is decreased in the uterus, an indication of the anoxic conditions present in female reproductive organs (Jeong *et al*., 2016). Cellular response in hypoxic conditions is mediated via HIF-1ɑ (Fadhillah *et al*., 2017). HIF-1ɑ regulates the expression of genes involved in adaptive response to hypoxic environments (Harvey *et al*., 2002). HIF-1ɑ is a transcription factor that has a supervisory role in the rate of VEGF gene expression (Wong *et al*., 2003). VEGF is regulated via HIF-1ɑ in hypoxic and normoxic circumstances (Pan *et al*., 2015). Our study showed that ovarian stimulation with gonadotropins exacerbated hypoxic conditions. No significant increases were seen in HIF-1ɑ gene expression, while a recent in vitro and in vivo study indicated that hCG led to the upregulation of HIF-1ɑ gene expression in luteinized granulosa cells (Fadhillah *et al*., 2017). Increased HIF-1ɑ gene expression led to increased angiogenesis via VEGF gene expression induction. Our data indicated that increases in HIF-1ɑ gene expression in the case groups caused increases in VEGF gene expression. HIF-1ɑ is affected by gonadotropins and can regulate VEGF mRNA expression in the luteal cells in vitro. Hypoxia is an important factor in the development of the vascular system during luteal development and results from HIF-1ɑ induction in luteal cells (Pan *et al*., 2015). Hypoxia also leads to the synthesis of P4 by granulosa cells during early corpus luteum formation (Fadhillah *et al*., 2014). Our data on HIF-1ɑ gene expression indicated that treatment with P4 in Case Group II yielded lower HIF-1ɑ gene expression levels than in Case Group І. It appears that excess P4 inhibits HIF-1ɑ gene expression and consequently decreases the rate of VEGF gene expression, as seen in Case Group II.

A potential bias in our study is the coordination of menstruation cycles of all included subjects and the estimation of implantation time. Pseudopregnancy helped us to overcome this limitation.

## CONCLUSION

Ovarian stimulation with P4 administration might increase ovarian angiogenesis. Although P4 treatment after ovarian stimulation causes slight increases in the rate of angiogenesis, it might improve angiogenesis throughout the ovarian tissue and yield better conditions for the establishment of pregnancy.
